# *Pulque*, a Traditional Mexican Alcoholic Fermented Beverage: Historical, Microbiological, and Technical Aspects

**DOI:** 10.3389/fmicb.2016.01026

**Published:** 2016-06-30

**Authors:** Adelfo Escalante, David R. López Soto, Judith E. Velázquez Gutiérrez, Martha Giles-Gómez, Francisco Bolívar, Agustín López-Munguía

**Affiliations:** ^1^Departamento de Ingeniería Celular y Biocatálisis, Instituto de Biotecnología, Universidad Nacional Autónoma de MéxicoCuernavaca, Mexico; ^2^Departamento de Biología, Facultad de Química, Universidad Nacional Autónoma de México, Ciudad UniversitariaCiudad de México, Mexico; ^3^Vagabundo CulturalAtitalaquia, Mexico

**Keywords:** *pulque*, *aguamiel*, maguey, lactic acid bacteria, *Saccharomyces cerevisiae*, dextran, fructans, probiotics

## Abstract

*Pulque* is a traditional Mexican alcoholic beverage produced from the fermentation of the fresh sap known as *aguamiel* (mead) extracted from several species of *Agave* (maguey) plants that grow in the Central Mexico plateau. Currently, *pulque* is produced, sold and consumed in popular districts of Mexico City and rural areas. The fermented product is a milky white, viscous, and slightly acidic liquid beverage with an alcohol content between 4 and 7° GL and history of consumption that dates back to pre-Hispanic times. In this contribution, we review the traditional *pulque* production process, including the microbiota involved in the biochemical changes that take place during *aguamiel* fermentation. We discuss the historical relevance and the benefits of *pulque* consumption, its chemical and nutritional properties, including the health benefits associated with diverse lactic acid bacteria with probiotic potential isolated from the beverage. Finally, we describe the actual status of *pulque* production as well as the social, scientific and technological challenges faced to preserve and improve the production of this ancestral beverage and Mexican cultural heritage.

## Introduction

The role of maize in the origin of humans as described in the *Popol Vuh*, the sacred Maya book, together with the betrayal of the Toltec god *Quetzalcoatl* by *Tezcatlipoca* -the omnipresent god of the night who sees everything- are the two favorite stories of Mesoamerican mythology. *Quetzalcoatl* was ruined and had to exile after a ridicule behavior due to an excess of *pulque* intake. Both maize and *pulque* were key in the cosmological vision in Mesoamerica: while maize was linked to their origins, *pulque* was associated to their destiny, the *Temoanchan*, or lost Paradise, inhabited by several gods, where humans were created and *pulque* invented. Both Quetzalcoatl and *Mayahuel* -the Mexican nurturing mother- came to Earth to sing and dance to escape from paradise and to adopt the form of tree branches. However, they were punished by *Mayahuel*'s grandmother who was a *tzitzimitl* -a darkness being- who, together with other *tzitzimime* destroyed the branch where *Mayahuel* was hiding. *Quetzalcoatl*, whose branch was not destroyed, buried *Mayahuel* with great sadness. The first agave plant grew in the place where *Mayahuel* was buried (Gonçalves de Lima, 1956; Anawalt, 1998; Ramírez, 2002).

However, the *Agavaceae* Family is very much older than pre-hispanic mythology, its origin dating back to about 10 million years ago (Good-Avila et al., 2006). Agave is a proliferous Family with nine known genera, comprising 300 species, most of them still present in Mexico. Agaves belong to the Amarilidaceas order and are endemic to Mexico. A restricted number of species are devoted to *pulque* including *A. atrovirens, A. americana, A. salmiana*, and *A. mapisaga* (Table 1; Alfaro Rojas et al., 2007; Mora-López et al., 2011).

**Table 1:** **Agave species used for ***aguamiel*** extraction and ***pulque*** production**.

**Name**	**Accepted name according to the Plant List web site^a^**	**Comments**	**References**
*A. atrovirens* Kraw ex Salm-Dyck	Accepted	Cultured mainly in the states of Mexico, Tlaxcala, Hidalgo y Puebla	Alfaro Rojas et al., 2007
*A. atrovirens* var. *salmiana* (Otto ex Salm-Dyck) Maire and Weiller	Synonym *A. salmiana* Otto ex Salm-Dyck	Cultured mainly in the states of Mexico, Tlaxcala, Hidalgo y Puebla	Alfaro Rojas et al., 2007
*A. americana* L	Accepted	Cultured mainly in the states of Mexico, Tlaxcala, Hidalgo y Puebla	Alfaro Rojas et al., 2007
A. *mapisaga* Trel	Accepted	Include 13 variants. Cultured mainly in the states of Mexico, Tlaxcala, Hidalgo y Puebla	Alfaro Rojas et al., 2007; Mora-López et al., 2011
*A. salmiana* var *angustifolia* A. Berger	Accepted	Cultured mainly in the states of Mexico, Tlaxcala, Hidalgo y Puebla	Alfaro Rojas et al., 2007; Mora-López et al., 2011
*A. salmiana* var *ferox* (K. Koch) Gentry|	Accepted	Include three variants	Mora-López et al., 2011
*A. salamina* var *salmiana*	Unresolved name	The most diverse group including 31 variants	Mora-López et al., 2011

a *The Plant List (2010). Version 1*.

The ancient Aztecs knew pulque as m*etoctli* (from nahuatl language *metl* = agave or maguey, and *octli* = wine) agave wine, or *iztacoctlli* (from *izac* = white and *octli* = wine) white wine, or *poliuhquioctli* (from *poliuhqui* = spoiled or rotted and *octli* = wine) the spoiled beverage with unpleasant odor and flavor. It is probably from *poliuhquioctli*, that the Spanish conquerors designated as *pulque*, the freshly fermented agave beverage (Gonçalves de Lima, 1956; Sahagún, 1999). *Pulque* is a milky white, viscous, and slightly acidic beverage whit an alcoholic content which depends on several factors but usually between 4 and 7° GL, produced by spontaneous fermentation of *aguamiel*, the sugary sap extracted from the *Agave* species mentioned above (Secretaría de Economía, 1972b). According to Fray Bernardino de Sahagún, in his “Historia General de las Cosas de Nueva España,” numerous gods were involved in the *Mayahuel*'s gift to humanity. Among others, he mentions *Ometochtli* who for the Aztecs was also the god of drunkenness, also associated with plant fertility and the wind. He ruled over the 400 *Centzontochtli*, or God rabbits of drunkenness, such as *Patecatl*, who knew how to mix *aguamiel* with plant roots, *Cuatlapanqui* (the “head- opener”) or *Papaztac* (the “nervous one”), among many others to whom the drunken and intoxicated were sacrificed (Gonçalves de Lima, 1956; Anawalt, 1998; Sahagún, 1999; Ramírez, 2002).

While most documents place the most probable origin of *pulque* in the ancient Otomi civilization toward the year 2000 BC, archeological evidence indicates that hunters and gatherers used maguey thousands of years ago (Jennings et al., 2005; Valadez-Blanco et al., 2012). Recent organic evidence shed new light on *pulque* history. In effect, although chemical components of this alcoholic beverage are water-soluble, limiting their conservation, hydrophobic lipids of food residues are more stable, Correa-Ascencio et al. (2014), applied a novel lipid biomarker approach to detect bacterial hopanoids derived from the widely recognized *pulque* fermenting bacteria *Zymomonas mobilis* as a *pulque* marker in more than 300 potsherds. The authors using this methodology were able to demonstrate for the first time the use of ceramic vessels to contain *pulque* in the locality of La Ventilla around 200–550 AD, at the height of Teotihuacan's culture. The presence of hopanes as bacterial markers of *pulque*, demonstrate that this beverage was produced in the ancient city of Teotihuacan and opens a new avenue of research for a systematic analysis to establish the level and intensity of *pulque* production and consumption in this culture (Correa-Ascencio et al., 2014).

During the height of the Aztec culture, *pulque* was produced and consumed preponderantly in religious and sacred rituals. It was restricted to the common citizens, with strict rules limiting its consumption. Excessive consumption was severely punished, in some cases including the capital punishment, even for priests. Upon the fall of the Aztec empire, *pulque* lost its religious significance gradually and became a food beverage and a popular intoxicant (Gonçalves de Lima, 1956; Ramírez et al., 2004; Ramírez Rodríguez, 2004). During the Spaniard Colony (1521–1821), *pulque* production was one of the main economic activities, and the most popular alcoholic beverage, resulting in the flourishment of *Haciendas pulqueras* (large farms dedicated to the cultivation of agave, *pulque* production, and commercialization), mainly in the central Mexican Plateau including the actual states of Hidalgo, Tlaxcala, Puebla, Morelos, Michoacán, and Querétaro. Interestingly, the production process remained practically unchanged since the Spaniard conquest and during Colony (Crist, 1939; Wilson and Pineda, 1963; Ramírez Rancaño, 2000). By 1629–1786, before the Mexican Independence War, *pulque* production and consumption was forbidden as it became a major health and social problems among the Indians. However, the economic relevance of maguey during the Spaniard Colony forced the authorities in 1786 to end the prohibition period as, despite the ban, *pulque* production competed with European wines and sugar cane liquor controlled by Spaniards (Lorenzo Monterrubio, 2007).

At the end of the Independence War (1810–1821), the production of *pulque* by the *Haciendas pulqueras* recovered its economic relevance, particularly by the introduction of the railway for the transport of thousands of liters of the fermented beverage directly from the *Haciendas pulqueras* to the main cities including Mexico City. By the beginning of the twentieth-century *pulque* production reached about 500 million L/year. By 1905, it is estimated that 350,000 L of *pulque* were consumed only in Mexico City. After the Revolution Civil War (1910–1920), the production structure of the *Haciendas pulqueras* was destroyed as *pulque* and its associated economic activity were owned by *hacendados*, an important part of to the upper class. By the period between 1920 to mid-1930s, the fresh *pulque* production and transport to Mexico City flourished again. However, by 1935–1940, the production and consumption of *pulque* was seriously affected again by an official anti-alcoholic policy, a severe devastation of agave plantations and the consolidation of the beer as a popular alcoholic beverage (Gonçalves de Lima, 1956; Ramírez Rancaño, 2000; Jácome, 2003; Ramírez et al., 2004; Ramírez Rodríguez, 2004; Lappe-Oliveras et al., 2008; Escalante et al., 2012).

*Pulque* had its major success in the last decades of the nineteenth century when rich fortunes derived from its successful production in *haciendas* and transport by train to the central Mexico urban centers. Significant efforts to preserve *pulque* and to face the increasing demand for *beer* failed. This effort, as well as the diversification of the agave industry, were led in particular by Ignacio Torres Adalid, known as “El Rey del *Pulque*” (“The King of *Pulque*” (Ramírez Rancaño, 2000). A campaign against *pulque* after the Mexican Revolution during the Venustiano Carranza government since 1914 until 1920, forced the *hacendados* to leave the country. *Pulque* consumption was associated with “criminality and degradation of the Mexican race.” That was the beginning of the *pulque* agroindustrial twentieth century debacle. Nevertheless, by 1882 *pulque* was the main alcoholic beverage consumed in the country and one of the most important Mexican agroindustries by the end of the nineteenth century. A train transported daily hundreds of wood barrels containing *pulque* from more than 300 *haciendas* and *tinacales* mainly from the Eastern states of Hidalgo and Tlaxcala, then rich region thanks to their “crops of the century” (maguey) and “white gold” (*pulque*) productivity (Parsons and Darling, 2000; Ramírez Rancaño, 2000; Jennings et al., 2005). Several factors have been mentioned to explain *pulque*'s decline, among others the fact that *pulque* could not cope with the introduction of a competing alcoholic beverage: *beer*.

Despite the substantial differences in composition and organoleptic properties, probably the fact that *pulque* consumption dropped dramatically during the first decades of the twentieth century, besides the already mentioned campaign against consumption, was the lack of investment in science and technology. Interestingly, while consumers are now favoring traditional beers over the industrialized product, *pulque* consumers have no choice other than the traditional product which, in the context of the actual consumption trends, is now paradoxically, an advantage. The number of *pulquerías* offering *pulque* in Mexico City has considerably increased with more than 100 places offered to the consumer in internet pages, most of them of high quality (Ramírez Rancaño, 2000). The main production in Mexico is still the central state of Hidalgo where more than 260 million liters of *pulque* were produced in 2010, equivalent to 82% of the national production, followed by Tlaxcala with 13.3% and the State of Mexico with 2.68%, according to unofficial sources. As far as the National Institute of Statistics (INEGI), beer is described as responsible in 2014 of 1.2% of the total bulk manufacturing, while *pulque* was 0.0022% (INEGI, 2016). Other sources such as the “Encuesta Nacional de Adicciones 2011” (Instituto Nacional de Salud Pública, 2011) estimates that beer is consumed by 50 and 30% of the male and female population respectively, while other fermented beverages like *pulque* are consumed by only 4.4% of the population.

## Traditional production of *pulque*

The main process of *aguamiel* extraction and *pulque* fermentation remains practically unchanged since pre-Hispanic times (Parsons and Darling, 2000; Jennings et al., 2005). Agave plants are relatively easy to cultivate as propagation is mainly carried by transplanting young off springs (called *matecuates* or *hijuelos*) from adult plants after a 7–25 years maturation cycle. Nevertheless, agave seeds cultivation has been an alternative for maguey propagation since pre-Hispanic times (Parsons and Darling, 2000). Agave plants are grown in specific agave plantations known as *magueyeras* where the trasplanted young *matecuates* are arrayed in parallel rows known as *melgas* or *metepnatle* (maguey wall) (Parsons and Darling, 2000; Ramírez Rancaño, 2000; Jácome, 2003). Agave plantations are located away from tall trees to avoid plant competence for light, water, and soil nutrients. Natural fertilization of agave plantations is self-provided by recycling naturally degraded agave plants or by the addition of agave ashes dispersed around the growing plants.

**Figure 1:**
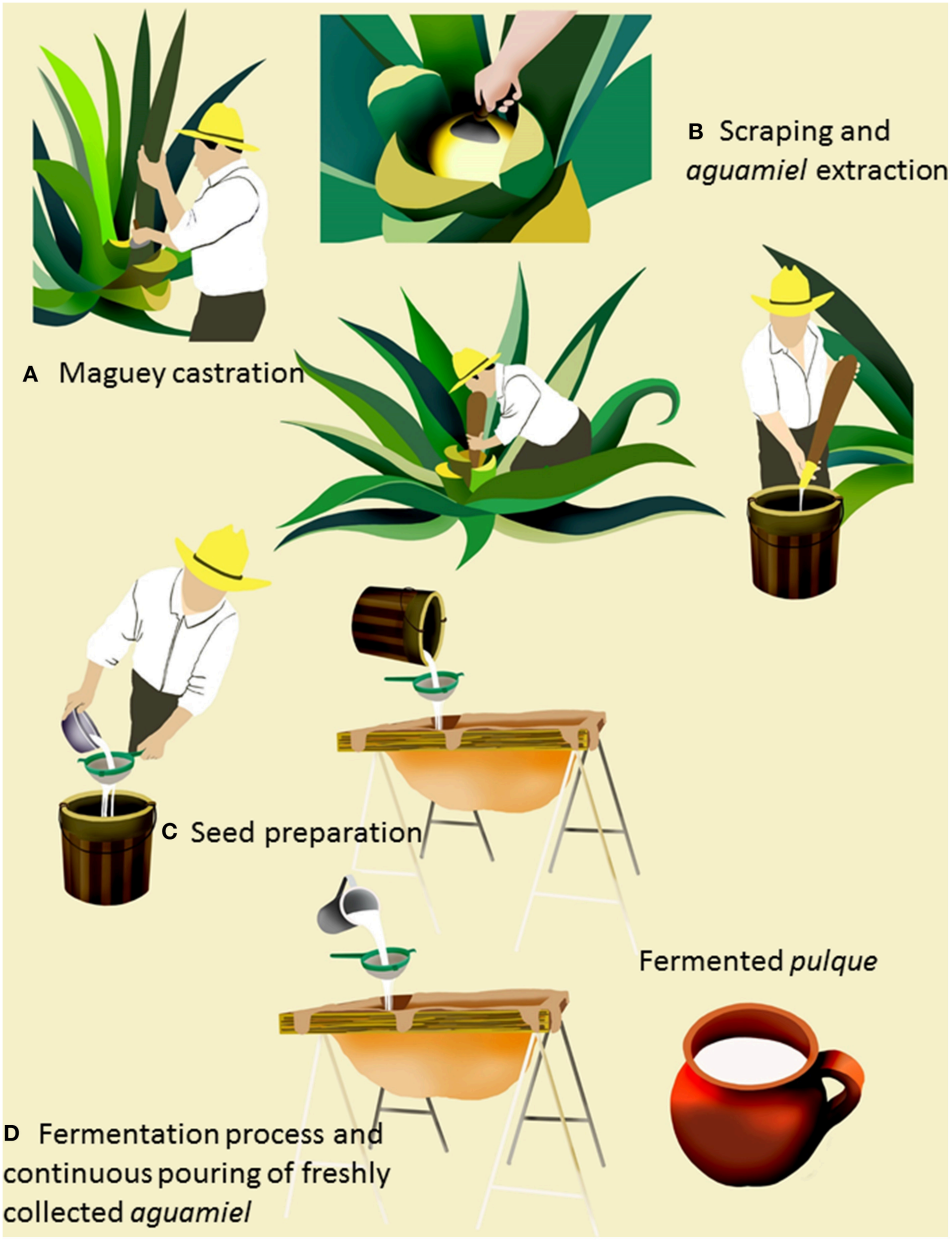
**Traditional ***pulque*** elaboration process**. The traditional process involves four common steps: **(A)** Castration of the mature plant by cutting the floral bud and make the pit (*cajete*). **(B)** Pit scraping to promote *aguamiel* accumulation and sap extraction. **(C)** Seed preparation. **(D)** Fermentation. For details of the castration process see Supplementary Files 1, 2.

*Aguamiel* extraction and *pulque* elaboration are performed traditionally by the *tlachiquero*, who has a deep knowledge of the biology and care of the maguey species used for production. The process starts with the selection of mature plants from 6 to 15 years old and comprises four common steps with slight variations across producing zones (Crist, 1939; Wilson and Pineda, 1963; García-Garibay and López-Munguía, 1993; Parsons and Darling, 2000; Jennings et al., 2005): (1) castration, (2) pit scraping and *aguamiel* extraction, (3) seed preparation, and (4) fermentation (Figure 1).

### Maguey castration

For this operation, selected mature plants are castrated by destroying the embryonic floral peduncle that surrounds the floral bud (*quiote*). During this operation, the central leaves of the plant (*meloyote* or heart), from which the flower rises are eliminated using a pointed and sharp instrument, leaving a cavity (known as *cajete*) in the center of the plant (Jennings et al., 2005). The cavity is covered with a large stone or with agave leaves to protect it from animals and the environmental conditions. A maturation period follows castration and varies from 3 months to 1 year (Crist, 1939; Wilson and Pineda, 1963; García-Garibay and López-Munguía, 1993; Parsons and Darling, 2000; Jennings et al., 2005).

The castration process varies among producing regions: in the production region of Huitzilac (Morelos state), the cavity is digged without eliminating the central leaves, and the floral bud is cut off after the maturation process. The precise moment for castration is the *thachiquero* responsibility to avoid floral budding. If the inflorescence grows, the plant will never produce *aguamiel*. Moreover, early castration will result in a reduced volume of poor quality *aguamiel* production. Traditionally, some hints used by the *tlachiquero* to select mature plants are the abundance of leaves, the thinness of *meloyote*, and the surrounding leaves, which are also spikeless and adopt a lighter green tone. A detailed video showing the castration process and the instruments used is available in Supplementary Files 1, 2 (Crist, 1939; Wilson and Pineda, 1963; García-Garibay and López-Munguía, 1993; Parsons and Darling, 2000; Jennings et al., 2005).

### Scraping and *aguamiel* extraction

**Figure 2:**
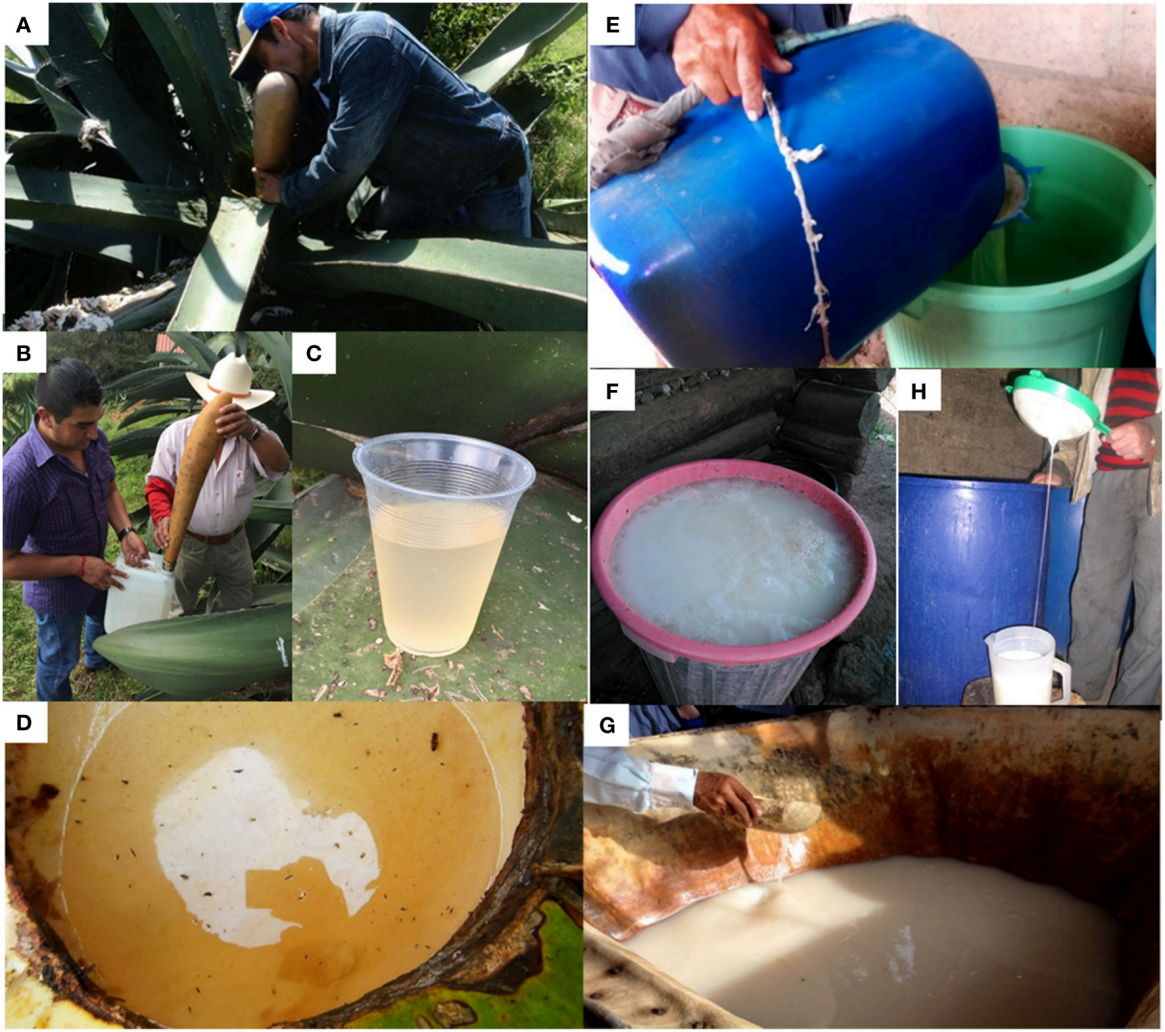
*****Aguamiel*** extraction from producing maguey, transportation to the ***tinacal*** and fermentation process**. **(A)** *Tlachiquero* extracting freshly *aguamiel* with an *acocote* (Hidalgo state). **(B)** *Aguamiel* is transferred into a plastic container for transportation to the *tinacal* (Morelos state). **(C)** Freshly collected *aguamiel* appearance (Morelos state). **(D)** *Aguamiel* accumulated in *cajete* previous to the twice-daily extraction (Hidalgo state). **(E)** *Aguamiel* pouring into a plastic vat for seed preparation (Hidalgo state). **(F)** Fermented *pulque* in a plastic vat (Hidalgo state). **(G)** Fermented *pulque* in a traditional leather vat (Hidalgo state). **(H)** Serving *pulque* for direct consumption from the fermentation vat (Tlaxcala state). Note the characteristic filament associated to final product viscosity.

Fresh *aguamiel* is a lightly cloudy, thick, very sweet, fresh-plant flavored and neutral to slightly acid sap. By scraping the *cajete*'s wall the sap outflow is induced, so *aguamiel* flows and accumulates in the cavity. This operation is performed by the *tlachiquero* using a scraping tool (Crist, 1939; Wilson and Pineda, 1963; García-Garibay and López-Munguía, 1993; Parsons and Darling, 2000; Jennings et al., 2005). The accumulated sap is extracted twice a day (usually at daybreak and dusk) by oral suction using a dried gourd (*Lagenaria siceraria*) known as *acocote*. After each *aguamiel* collection, the walls of the cavity are scraped again to maintain the sap flow induction. Freshly collected *aguamiel* is stored in plastic containers and transported to specific vats where the main fermentation takes place (Figure 2). A mature agave plant may produce *aguamiel* from 3 to 6 months until the plant dies, depending on the frequency of the scraping process. On a daily basis, the plant yields 4–6 L of *aguamiel* with a maximum average production of around 1000 L in its production lifetime (Crist, 1939; Wilson and Pineda, 1963; García-Garibay and López-Munguía, 1993; Parsons and Darling, 2000; Ramírez Rancaño, 2000; Jennings et al., 2005).

### Seed preparation

This operation refers to the production of starting material (inoculum) for the fermentation of freshly collected sap in a new container. For this purpose, around 2 L of fermented *pulque* are placed in a ~20 L vat made of clay, glass, wood, plastic or fiberglass, were fresh, high-quality *aguamiel* is poured. A spontaneous fermentation starts at room temperature until a characteristic alcoholic, and acetic taste develops or until a white layer -called *zurrón*- is formed on the surface, a process that usually takes from 1 to 4 weeks, depending on the season). Finally, the *tlachiquero* transfers the fermented product (seed) to one or more clean vats where *pulque* fermentation will takes place once freshly collected *aguamiel* is added (Crist, 1939; García-Garibay and López-Munguía, 1993; Parsons and Darling, 2000; Jennings et al., 2005; Escalante et al., 2012).

### *Pulque* fermentation

Fermentation takes place in vats usually made of cow-leather, glass-fiber, plastic or wood barrels located either in closed rooms known as *tinacal* or in specific open spaces (Figure 2). Freshly collected *aguamiel* is filtered to separate insects or any large object and poured into the vat, where the seed was previously transferred. The fermentation time varies strongly depending on *aguamiel* quality, seed maturity, season and producing region, among other factors. It usually lasts from 3 to 6 h, but overnight or even extended periods of time (e.g., 3–12 days) are not uncommon (Crist, 1939; Parsons and Darling, 2000; Ramírez Rancaño, 2000; Jennings et al., 2005).

Mexican norm NMX-V-022.1972 defines the sensorial properties required for the fresh collected sap or *aguamiel* used for *pulque* fermentation as a translucent, light amber-colored, sweet, fresh-flavored and lightly acid liquid with characteristic flavor and odor. Based on their physicochemical properties this norm defines two types of *aguamiel*. Type I or high-quality *aguamiel* and Type II, poor quality or slightly acid *aguamiel*. As for the alcohol content, Mexican norm NMX-V-037-1972 defines the alcoholic content of *pulque*. According to this norm, *pulque* is a beverage with low alcoholic content, not-clarified, of white color, acid, and viscous texture. The norm defines two types of *pulque*, Type I or *pulque* for seed (Section Biochemistry of the Fermentation) and “*puntas*” and Type II or commercial *pulque*. The requirements specified for *aguamiel* and *pulque* in norms NMX-V-022.1972 and NMX-V-037-1972 are presented in Table (Secretaría de Economía, 1972a,b).

Despite the Mexican norm NMX-V-037-1972 defined the desirable physicochemical properties of bulk *pulque* for direct consumption, particularly for density, pH (3.5–4.2), and alcohol degree (4–9%) (Table 2; Secretaría de Economía, 1972b), during traditional production of *pulque* the degree of fermentation varies according to the producer and is considered adequate when a characteristic alcohol, acetic notes, and texture (viscosity) is reached. Fermented *pulque* is withdrawn from the vat and consumed either natural or *curado*, as it is known when mixed with macerated fruits, vegetables, nuts or spices (Parsons and Darling, 2000; Ramírez Rancaño, 2000; Jennings et al., 2005; Lappe-Oliveras et al., 2008; Escalante et al., 2012). Sometimes, particularly when the fermentation yields a low-quality *pulque* (e.g., with low viscosity or off flavors), the *tlachiquero* adds plant roots, herbs or pieces of agave plants, a practice known as *cardón*, to improve the fermentation process (Parsons and Darling, 2000; Jennings et al., 2005).

**Table 2:** **Physicochemical characteristics of ***aguamiel*** and ***pulque*****.

**Characteristic**	***Aguamiel***				**References**
	**Type I**		**Type II**		
	**Minimum**	**Maximum**	**Lower to**		
pH	6.6	7.5	4.5		Secretaría de Economía, 1972a
Density (°Bé)	5	7	4.5		
Refractive index (immersion, 20°C)	59	100	27		
Total solids^a^	13	17	7		
Total reducing sugars^a^ (as glucose)	8	12	6		
Direct reducing sugars^a^ (as glucose)	2	3	3		
Gums^a^ (as glucose)	2	6	0.2		
Proteins^a^	300	600	100		
Ashes^a^	300	430	100		
Total acidity^a^ (as lactic acid)	0.9	1.03	4		
	***Pulque***				
	**Type I**		**Type II**		
	**Minimum**	**Maximum**	**Minimum**	**Maximum**	
Refractive index (immersion, 20°C)	32	35	25	ND	Secretaría de Economía, 1972b
Refractive index (Abbé, 20°C)	1.3390	1.3406	1.3365	1.3380	
pH	>3.7	4.2	3.5	4	
Total acidity^a^ (as lactic acid)	0.4	0.75	0.4	0.7	
Total reducing sugars^a^ (as glucose)	0.1	0.8	0.2	0.5	
Alcoholic degree (%/vol)	6	9	4	6	

a *mg/100 mL, ND, non-defined. °Bé, Baumé degrees*.

## Microbiology and biochemistry of the fermentation

### Toward the definition of an essential microbiota responsible for *pulque* fermentation

*Pulque* fermentation is a batch non-stirred process, performed under non-aseptic conditions. The microorganisms involved in the fermentation are those naturally occurring during sap accumulation in the *cajete* cavity in maguey and those incorporated during collection, transport, seed preparation and manipulation (Lappe-Oliveras et al., 2008; Escalante et al., 2012). Earlier studies on the microbiology of *pulque* performed by Sanchéz-Marroquín by 1950's reported the presence of homo- and heterofermentative LAB identified as *Lactobacillus* sp., *Leuconostoc mesenteroides*, and *L. dextranicum*, the yeast *Saccharomyces cerevisiae* (identified as *S. carbajali*) and the α-Proteobacteria *Zymomonas mobilis* (identified as *Pseudomonas lindneri*) (Sánchez-Marroquín and Hope, 1953).

These microorganisms develop three distinctive metabolic products during *pulque* fermentation: lactic acid produced by *Lactobacillus* sp. and *Leuconostoc* sp. which conduct the acid fermentation, ethanol resulting from the alcoholic fermentation and synthesized mainly by *S. cerevisiae* and *Z. mobilis*, and the extracellular polysaccharides (EPS), which include dextrans and fructans produced from sucrose by glycosyltransferases from *Leuconostoc* sp. and *Z. mobilis* (Sánchez-Marroquín and Hope, 1953; Lappe-Oliveras et al., 2008; Escalante et al., 2012). Due to this complex fermentation process, *pulque* is considered an acid and viscous alcoholic beverage. Sánchez-Marroquín et al. (1957), used isolated strains of the species mentioned above in a mixed inoculum, as a starter for a controlled fermentation of *aguamiel*. The Sánchez-Marroquín group was able to obtain a fermented beverage with similar organoleptic and physicochemical characteristics of the fermented product regarding flavor, aroma, alcohol content, acidity, and viscosity, suggesting the essential role of these microorganisms in traditional *pulque* properties (Sánchez-Marroquín et al., 1957).

Further studies on the microbiology of *pulque*, allowed the identification of a wider bacterial and yeast diversity. This diversity has been classified according to the microorganisms' main metabolic traits as (i) acid producing bacteria, including LAB and acetic acid bacteria (AAB); (ii) alcohol-producing microorganisms, including *S. cerevisiae* and *Z. mobilis*, (iii) dextran-producing bacteria (*L. mesenteroides*), and (iv) putrefactive microorganisms (Table 2). Interestingly, microorganisms involved in the four fermentative processes of *pulque* fermentation have been systematically isolated in *pulque* samples of different regions around the central Mexican Plateau (Escalante et al., 2004; Lappe-Oliveras et al., 2008). Regarding yeast diversity in *pulque, Saccharomyces*, and non-*Saccharomyces* species have been identified and proposed as essential fermentative yeast responsible for the production of ethanol, amino acids, vitamins, and volatile flavor compounds participating in the sensorial properties of the beverage (Lappe-Oliveras et al., 2008). Additionally, diverse killer and killer-resistant yeasts were isolated from *aguamiel* and *pulque*, some of them with a remarkable alcohol tolerance (Estrada-Godina et al., 2001) (Table 2).

Analysis of the bacterial diversity of *pulque* samples of different geographical origins (Estado de Mexico, Hidalgo, and Morelos states) as determined by 16S rDNA clone libraries was reported by Escalante et al. (2004). These authors reported the identification of an even wider diversity including non-previously reported bacteria. Interestingly, this study allowed to conclude that the bacterial diversity present among *pulque* samples was dominated by LAB, particularly *Lactobacillus acidophilus* (homofermentative LAB), corresponding to ~60–85% of total 16S rDNA clones analyzed for each *pulque* sample. Other clones identified as *L. mesenteroides* ranging from ~0.5 to 25% of total clones analyzed for each sample. *Z. mobilis* was detected in low amounts only in two samples, and 16S rDNA clones identified as the AAB *Acetobacter pomorium* and *Gluconobacter oxydans* (~33% of detected clones) were detected only in one sample. These results allowed defining the common bacterial diversity in *pulque* samples of different geographical origin, as well as a bacterial diversity specific of a given region (Escalante et al., 2004).

### Assessment of the changes in the bacterial community during the fermentation of *pulque*

The dynamics of bacterial diversity was studied in the laboratory with fresh *aguamiel* and *pulque* collected from Huitzilac, Morelos state by Escalante et al. (2008), using a polyphasic approach, including the isolation of LAB, aerobic mesophiles, and 16S rDNA clone libraries from total DNA extracted from fresh collected *aguamiel* used as substrate, after inoculation with previously produced *pulque* and followed by 6-h fermentation. Freshly collected *aguamiel* contained a count of 1.3 × 10^7^ CFU/mL of total aerobic mesophilic bacteria (AMB), 3.2 × 10^9^ CFU/mL of total LAB, and 3.1 × 10^4^ CFU/mL of total yeasts. These results revealed the presence of a major microbial content associated to the accumulated sap in the maguey cavity (Escalante et al., 2008).

These authors also reported that total microbial counts determined after mixing fermented *pulque* with freshly collected *aguamiel* (initial fermentation time = 0 h) resulted in an increase of yeasts to 8.8 × 10^6^ CFU/ml. After three h of fermentation, total yeasts further rose to 1.4 × 10^7^ CFU/mL and remained constant until the end of the fermentation (1.9 × 10^7^ CFU/mL). Total counts of both bacterial groups at the beginning of the fermentation were 1.2 × 10^7^ CFU/mL for total AMB and 1.5 × 10^8^ CFU/mL for LAB. By the end of the fermentation, total counts of both bacterial groups remained relatively constant as reached 3.5 × 10^7^ CFU/mL and 1.5 × 10^8^ CFU/mL, respectively (Escalante et al., 2008).

The microbial diversity identified in *aguamiel* was composed mainly by LAB including *L. mesenteroides, L. kimchi, L. citreum* and in minor proportion *Lactococcus lactis*. The γ-Proteobacteria *Erwinia rapontici, Enterobacter* sp., and *Acinetobacter radioresistens* were the second most abundant bacterial group detected in agave sap. As the identified γ-Proteobacteria are naturally distributed microorganisms in diverse environments such as freshwater, soil, and vegetable surfaces, it may be possible to suppose that these bacteria are a contaminant incorporated to the *sap* during its accumulation in the *cajete*, or during the extraction and handling procedures (Escalante et al., 2008). Although Escalante et al. (2008) did not report the detection of lactobacilli in *aguamiel*, the isolation of *Lactobacillus brevis* and *L. collinoides* from agave sap samples collected from Huitzilac, Morelos state, was described in a recent publication (Reyes-Naya et al., 2016).

**Figure 3:**
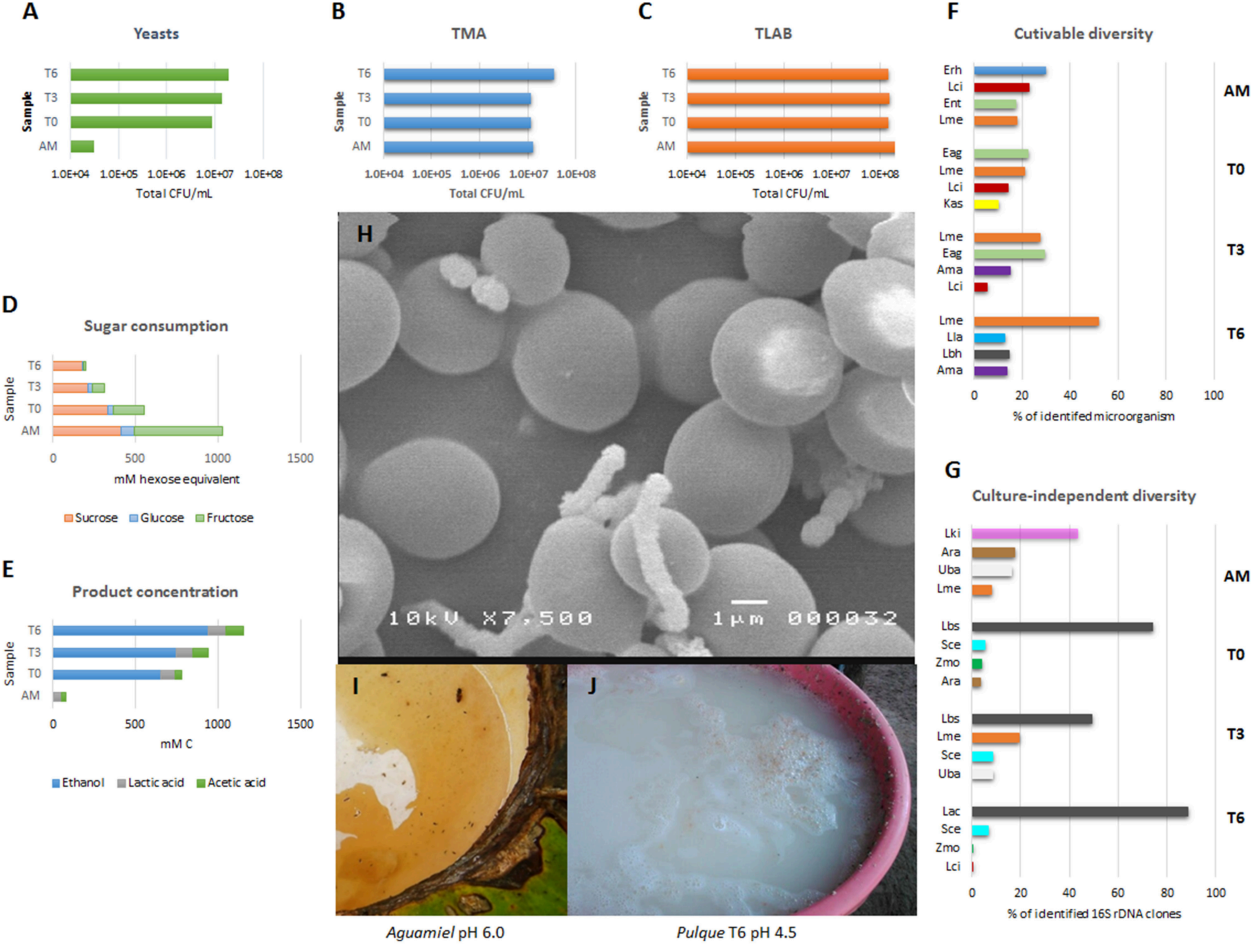
**Microbial, metabolic and physicochemical changes during ***pulque*** fermentation**. Proposed microbial, physicochemical and metabolic changes during *pulque* fermentation as described by Escalante et al. (2008). **(A)** Total CFU/mL counts for yeasts; **(B)** Total mesophilic aerobes (TMA); **(C)** LAB determined during 6 h fermentation in laboratory; **(D)** Sugar consumption expressed as mM hexose equivalent; **(E)** Fermentation products (ethanol, lactic acid, and acetic acid) expressed as mM C; **(F)** Cultivable diversity (% of four most abundant isolates); **(G)** Culture-independent diversity (% of four most abundant 16S rDNA clones); **(H)** Scanning electron micrograph corresponding to *pulque* fermentation after 6 h showing some yeast and short cocci chains (T6) (non-previously published photograph); **(I)** *Aguamiel* accumulated in *cajete*; **(J)** Fermented *pulque*. AM, *aguamiel*, T0, T3, and T6, the start of the fermentation, 3 and 6 h of cultivation, respectively. Ama, *Acetobacter malorum*; Ara, *Acinetobacter radioresistens*; Eag, *Enterobacter aglomerans*; Erh, *Erwinia rhapontici*; Ent, *Enterobacter* sp.; Kas, *Kluyvera ascorbata*; Lbh, homofermentative *Lactobacillus* sp.; Lbs. *Lactobacillus* sp.; Lac, *L. acidophilus*; Lla, *Lactococcus lactis*; Lme, *Leuconostoc mesenteroides*; Lci, *L. citreum*; Lki, *L. kimchi;* Sce, *Saccharomyces cerevisiae*; Zmo, *Zymomonas mobilis;* Uba, Uncultured bacterial clone.

The addition of freshly collected *aguamiel* to previously fermented *pulque* results in a considerable increase in the count of yeasts (~155% on total CFU/mL respect *aguamiel*). *L. kimchi* and *A. radioresistens* decreased, and *L. mesenteroides* remained relatively constant respect *aguamiel* (Escalante et al., 2008). Interestingly, after mixing *aguamiel* with *pulque* (T0), the most abundant microorganism detected was the LAB identified as *Lactobacillus acidophilus*. The γ-Proteobacteria *Enterobacter agglomerans*, and the α-Proteobacteria *Z. mobilis* and *Acetobacter malorum* were also detected but in low proportions in T0. Important physicochemical changes were observed in T0. After mixing fresh *aguamiel* and fermented *pulque*, the pH decreased from 6.0 to 4.5 in the mixture. Total sugars in *aguamiel* decreased 53.9%, and total carbon in fermented products detected in T0 (mainly as ethanol) increased 942.5% when compared to *aguamiel* (Escalante et al., 2008; Figure 3).

Microbial diversity present at T0 includes microorganisms in *aguamiel* and those from fermented *pulque* resulting in a microbial diversity composed by homo- and heterofermentative LAB, EPS-producing LAB, AAB, AMB, ethanol producing *Z. mobilis*, and yeasts. After 3 h of fermentation, diverse changes in the microbial diversity occurred despite the relatively constant total CFU/ml observed for LAB and total AMB. *L. acidophilus, L. mesenteroides*, and *E. agglomerans* were the most abundant bacteria; some others (both LAB and Proteobacteria) decreased or disappeared while yeast increased 102.9%. Also after 3 h, total sugars measured in T0 decreased 56%, and total carbon in fermented products (mainly ethanol) increased 120.7%. Finally, after 6 h of fermentation, the final microbial diversity was composed mostly by the homofermentative *L. acidophilus, L. mesenteroides. L. lactis* subsp. *lactis* and the α-Proteobacteria *A. malorum*. As a consequence of the microbial activity, after 6 h of fermentation, the final pH further decreased to 4.3, while 63.3% of the total sugar present after inoculation was consumed. Final fermentative products corresponded to 939.5 mM C as ethanol, 106.2 mM C as acetic acid, and 108 mM as lactic acid (Figure 3; Escalante et al., 2008).

### Biochemistry of the fermentation

**Figure 4:**
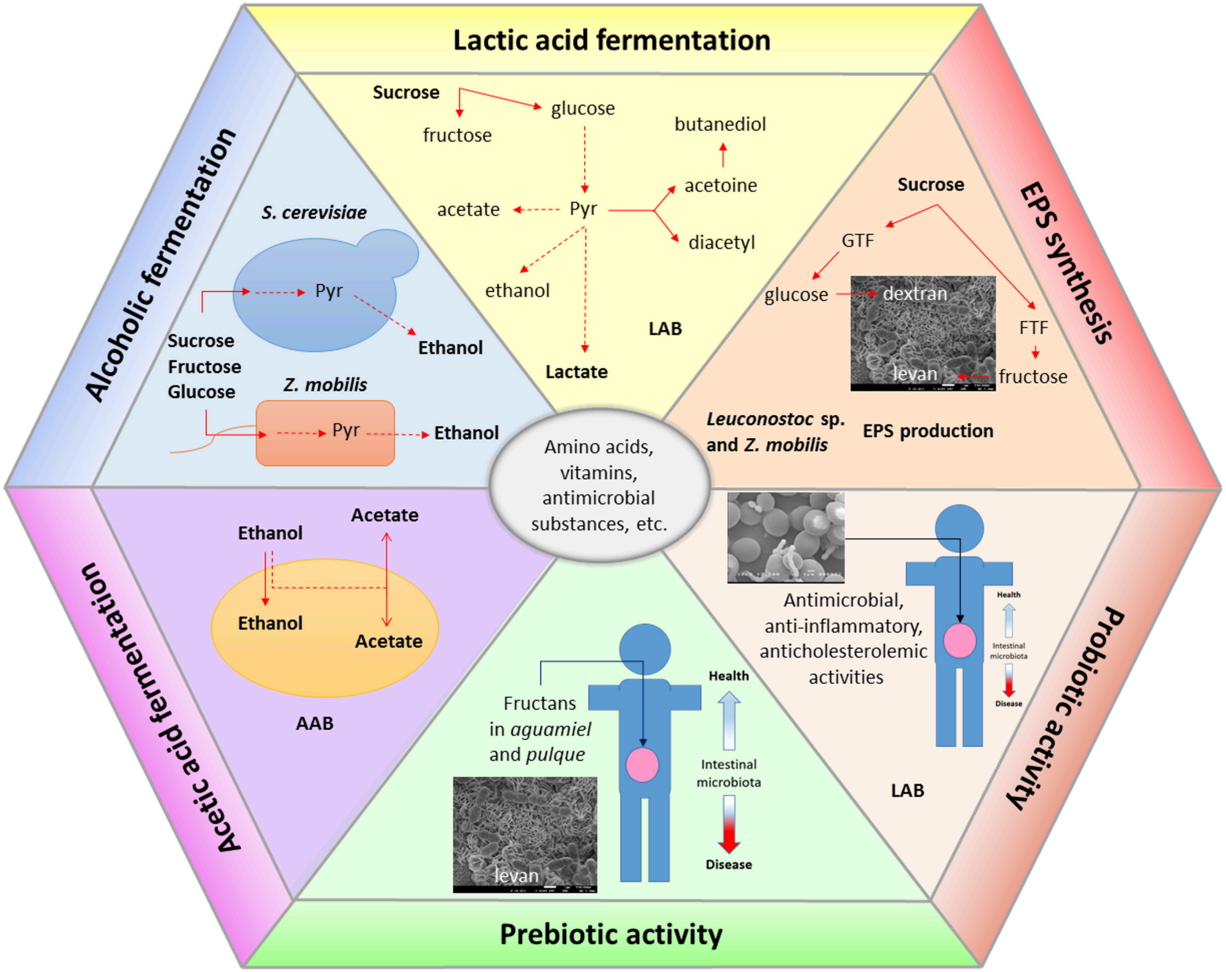
**Metabolic traits of main microbial groups present in ***aguamiel*** and during ***pulque*** fermentation**. Main metabolic traits comprise homo- and heterofermentative lactic acid metabolism by LAB. Production of ethanol by *Saccharomyces*, non-*Saccharomyces* yeasts, and *Z. mobilis*. Acetic acid metabolism. Extracellular polysaccharide synthesis resulting in the synthesis of dextran and levan polymers by *Leuconostoc* sp. and *Z. mobilis* (levan). Microorganisms and metabolic pathways involved in the amino acid production, vitamins, and some antimicrobial compounds remain to be determined. Functional properties such as prebiotic and probiotic activities are related to fructooligosaccharide content in *aguamiel* and *pulque* or produced by LAB such as *Leuconostoc* sp. Probiotic properties are related to diverse LAB identified as *Lactobacillus* sp. and *Leuconostoc* sp.

As already described, microbiological studies of *aguamiel* and *pulque* have revealed the presence of a complex bacterial and yeast diversity. The final sensorial properties of *pulque* are defined by the simultaneous development of the four fermentation types already described in Section Toward the Definition of an Essential Microbiota Responsible for Pulque Fermentation, which depend on the most abundant microorganisms present in *pulque*, also depending on its geographical origin (Figure 4):

- An acid fermentation performed mainly by homo- and heterofermentative LAB such as *Lactobacillus* and *Leuconostoc* (Sánchez-Marroquín and Hope, 1953; Sánchez-Marroquín et al., 1957; Escalante et al., 2004, 2008; Lappe-Oliveras et al., 2008), species involving the catabolism of available glucose to pyruvate by the Embden-Meyerhoff pathway and its subsequent conversion to lactic acid and other metabolic products such acetic acid, CO_2_, and ethanol (Carr et al., 2002).
- An alcoholic fermentation performed mainly by the yeast *S. cerevisiae* and in minor degree by the α-Proteobacteria *Z. mobilis* from sucrose, glucose, and fructose in *aguamiel*. *Z. mobilis* converts efficiently fermentable sugars to ethanol and CO_2_ by the Entner-Doudoroff pathway (Lau et al., 2010; Xiong He et al., 2014).
- The synthesis of EPS performed by *Leuconostoc* species including *L. mesenteroides* and *L. kimch*i resulting in the production of dextran and fructan exopolysaccharides from sucrose by enzymes such as glucosyl- and fructosyl-transferases, respectively (Chellapandian et al., 1998; Torres-Rodríguez et al., 2014). *Z. mobilis* is also a levan producer (Xiong He et al., 2014).
- An acetic acid fermentation performed probably by AAB such *Acetobacter* and *Gluconobacter* species (Escalante et al., 2004, 2008). AAB produce acetic acid as the main product through the oxidation of sugars, sugar-alcohols, and ethanol by the sequential activity of alcohol dehydrogenase and aldehyde dehydrogenase located in the outer membrane. *G. oxydans* catabolizes preferentially sugars and *Acetobacter* sp. in a minor proportion. Additionally, these bacteria produce gluconic acid and oxidize several organic acids including lactic acid to CO_2_ and water (Raspor and Goranovič, 2008).

The specific role of diverse microorganisms, particularly those identified as dominant in *aguamiel* and *pulque* fermentation in the production of essential amino acids, vitamins, and a variety of flavored volatile compounds remains a research subject (Figure 4).

## Functional properties of *aguamiel* and *pulque*

### Nutritional benefits associated with *pulque* consumption

According to the traditional pharmacopeia, *aguami*el and *pulque* consumption has been related to diverse nutritional and health-promoting benefits since Pre-Hispanic times despite the alcohol content of the fermented beverage (mild value ~4.8% ethanol) (Secretaría de Economía, 1972b; Backstrand et al., 2002). However, the first study directly reporting the health benefits of *pulque* consumption, is the successful treatment of scurvy in penitentiary inmates in 1887 in Puebla state, well before the discovery of vitamin C (Ramírez Rancaño, 2000). The first systematic study on the nutritional benefits of *pulque* consumption associated with a regular intake was carried out in the indigenous Otomí population of theValle del Mezquital (Hidalgo state) was performed by Anderson et al. (1946). Results obtained from the analysis to 100 adult consumers, under a 7 days' based diet, conclude that daily intake of *pulque* (up to 2 L) provides calories (12%), total protein (6%), thiamin (10%), riboflavin (24%), niacin (23%), vitamin C (48%), calcium (8%), and iron (51%). These results indicate that for this ethnic group, *pulque* consumption constitutes the second most important “food” in the diet after tortilla. Authors concluded that these results are relevant considering the marginal character of this indigenous population diet, highlighting the daily contribution of vitamin C trough *pulque* (Anderson et al., 1946).

Sánchez-Marroquín and Hope (1953), determined the main content of some vitamins in *pulque* (μg/100 mL of *pulque*) and found: 65.2 of pantothenic acid, 30.7 of thiamine, 21.6 of ρ-amino benzoic acid, 23 of pyridoxine, including also 19.6 (ng/100 mL of *pulqu*e) of biotin (Sánchez-Marroquín and Hope, 1953). Further studies on the nutritional benefits of *pulque* intake demonstrated that after maize tortillas and legumes, *pulque* was the third most important source of iron (non-heme form), ascorbic acid, riboflavin, and other B-vitamins. Additionally, *pulque* provides significant amounts of folate, steroidal saponins, many of them bioactive (Backstrand et al., 2002). Furthermore, *pulque* is a source of phytase which has been proposed to be produced by *Lactobacillus* species and *S. cerevisiae* present in *pulque*, resulting in an increased bioavailability of iron and zinc present in maize (Tovar et al., 2008). Regarding the amino acids content, it was found that *pulque* contains 0.27 g/100 *pulque* of crude protein. Detected amino acids (g/16 g of N), included Ile (4.04), Leu (8.65), Lys (1.76), Cys (1.59), Phe (6.45), Tyr (2.76), Thr (4.21), Trp (2.35), Val (5.12), and His (2.01) (Morales de León et al., 2005). The total content of protein and amino acids is substantially less than what the common myth in rural areas propose, which is that “*pulque* lacks one degree to have the benefits of meat.”

Studies on the relationship of iron status in a rural population from central Mexico highlands (Valle de Solís), performed in 125 non-pregnant women aged between 16 and 44 years old, assessed food intake during 12 months. Iron status determined after blood analysis showed higher plasma ferritin concentrations associated with significant intakes of non-heme iron and ascorbic acid. This study showed that better iron status correlated with significant *pulque* intake, an important source of non-heme iron and ascorbic acid, influencing the iron status of women from this rural zone. In this study, daily ethanol intake by *pulque* consumption was calculated using an average content of 47 g ethanol/L *pulque*; wich corresponds to the mean between 29 and 65 g/L (Backstrand et al., 2002).

The study of *pulque* intake in 70 expectant mothers from the Valle de Solís showed that 72.9% of women included in the study consumed *pulque* during pregnancy, and 75% continued consumption during the postpartum period as an important source of nutrients and energy. The consumption of 0.5 L of *pulque*, the amount commonly consumed by women in the research site supplied 24 g of ethanol, 9% of energy, 42.9% of ascorbic acid, 6.7 of thiamine, 5.9% of riboflavin, and 14.6% of iron of the Mexican Recommended Dietary Intake (RDI) during pregnancy. Results indicated that ascorbic acid intake from *pulque* was associated with a decrease in the risk of low ferritin and hemoglobin levels. The ethanol content in *pulque* was proposed to enhance iron absorption and to improve mother's daily iron intake. These authors showed the association between *pulque* intake during lactation and robust newborn growth, suggesting a beneficial effect of low *pulque* intake associated probably to the micronutrient content of the beverage. However, the study concludes that earlier intake of *pulque* during pregnancy and lactation was associated with poorer child height and weight (Backstrand et al., 2001, 2004).

### *Aguamiel* nutritional content and possible functional properties

Regarding *aguamiel*, the sap collected from *A. salmiana* ‘Gentry’ contains low amounts of crude fiber (0.57%), crude protein (0.69%) and a high level of nitrogen free extract (98.1%, corresponding to highly digestible carbohydrates). Mineral content analysis showed (in mg/L of *aguamiel*) 100 of N, 200 of Ca, 200 of P, 200 of Mg, 21.5 of Fe, 14.1 of Zn, 7.4 of Cu, and 19.9 of B. The consumption of 850 mL of *aguamiel* satisfy the daily human requirements of Fe and Zn, according to the Recommended Dietary Allowances or Adequate Intake (Silos-Espino et al., 2007).

The sap collected from *A. mapisaga* ‘Blanco’ contains (wt % in dry matter) 11.5% composed mainly of 75% of sugars (sucrose, fructose, glucose, and fructooligosaccharides), 0.3% of free amino acids (essential amino acids with exception of methionine), 3% of proteins, and 3% of ashes. Besides essential amino acid 26 mg/L of *aguamiel* of γ-aminobutyric acid (GABA) were identified (Ortiz-Basurto et al., 2008). These authors determined that *aguamiel* composition remain relatively stable throughout the production period (5 months), suggesting that the sap produced by *A. mapisaga* could be a stable substrate for a standardized *pulque* production processes.

*Agave* plants possess branched fructans (graminan) and graminan neoseries with two branches. One branch is attached to the fructosyl residue while the other is attached to the glucosyl unit of the sucrose molecule. These fructans have been designated as *agavins*, which are inulins with a complex mixture of structures and different degree of polymerization (DP) (Velázquez-Martínez et al., 2014). Due to the high fructan and fructooligosaccharide (FOS) content, agave extracts as well as the sap (consumed directly or concentrated) from different species, have been considered as an alternative source for prebiotic FOS syrups. This type of food additives has received increased attention due to its low glycemic index and, their demonstrated beneficial health effects such as improving calcium absorption in postmenopausal women, iron absorption, and, colon cancer prevention (García-Aguirre et al., 2009; Santos-Zea et al., 2016). *Aguamiel* from *A. mapisaga* “Blanco” contains inuline-type fructans (10.2% wt in dry matter) and glucooligosaccharides. The fructooligosaccharides identified up to now are highly branched, containing β-fructosyl units linked mainly by β1 → 2, but also β2 → 6 linkages (Ortiz-Basurto et al., 2008). Different extracts of *A. angustifolia* “Haw” agave have high molecular weight and branched fructans with the same structure regarding fructan linkages but different DP: high (3–60 fructose units), medium (2–40), and low (2–22) (Velázquez-Martínez et al., 2014).

*Agave* fructooligosaccharides have a demonstrated prebiotic function. In effect, several reports have demonstrated the *in vitro* growth promoting effects of diverse lactobacilli and bifidobacteria and well-known probiotic strains including *L. acidophilus, B. lactis, B. infantis, B. animals*, and *B. adolescentis*, some of them considered as predominant in human intestinal microbiota (Tripathi and Giri, 2014; Velázquez-Martínez et al., 2014; Castro-Zavala et al., 2015). As discussed above, *aguamiel* and *pulque* possess diverse well-documented nutritional traits; the main disadvantage of *pulque* remains its alcoholic content, which limits and restricts its promotion and consumption (Narro-Robles and Gutiérrez-Avila, 1997; Backstrand et al., 2001, 2004).

### Assessment of the probiotic potential of LAB isolated from *aguamiel* and fermented *pulque*

The isolation and assessment of the probiotic potential of LAB from non-dairy products for the formulation of health-promoting functional foods have been a trending activity (Tripathi and Giri, 2014). This type of products containing probiotic bacterial strains but based on juices, fruits, and cereals, offer significant advantages as an alternative to dairy-based functional products such as low cholesterol and the absence of dairy-allergenic substances (Soccol et al., 2012).

LAB detected as the most abundant bacteria in *pulque* such as *Lactobacillus acidophilus* and *L. plantarum* (Table 3), are proposed to play an important role also due to their antimicrobial activities. The natural resistance of these LAB to the final *pulque* pH and alcohol content, their abundance at the end of fermentation (Escalante et al., 2008), and the traditional application of *pulque* for the treatment of gastrointestinal diseases suggest that LAB involved in *pulque* fermentation are potential probiotic candidates.

**Table 3:** **Microbial diversity detected in ***aguamiel*** and during ***pulque*** fermentation**.

**Bacteria**	**Yeasts/Fungi**	**Remarkable metabolic traits defining sensorial properties of *aguamiel* or *pulque***	**References**
*Lactobacillus* sp. *Leuconostoc mesenteroides, L. dextranicum Zymomonas mobilis*	*Saccharomyces cerevisiae*	Essential microorganisms responsible for acid (lactic acid), alcoholic and production of EPS	Sánchez-Marroquín and Hope, 1953; Sánchez-Marroquín et al., 1957
	Yeasts isolated from *aguamiel: Candida lusitaneae, Klyuveromyces marxianus* var *bulagricus* (+)*, S. cerevisiae* Yeast isolated from *pulque*: *C. valida* (+)*, S. cerevisiae* (*chevalieri*), *S. cerevisiae* (*capensis*), *K. marxianus* var *lactis* (+)	Several isolates of *C. valida, S. cerevisiase* (*chevalier*) isolated from *pulque* were able to resist to >10% of alcohol. Potential relevance in ethanol production during the fermentation and resistance to killer toxins	Estrada-Godina et al., 2001
*Acetobacter aceti, A. aceti* subsp. *xylinus, Bacillus simplex, B. subtilis, Cellulomonas* sp., *Escherichia* sp., *Kokuria rosea, Lactobacillus* sp., L. *delbrueckii, L. vermiforme, Leuconostoc* sp., *L. mesenteroides* subsp. *dextranicum*, L. *mesenteroides* subsp. *mesenteroides, Macrococcus caseolyticus, Micrococcus luteus, Sarcina* sp., *Z. mobilis* subsp. *mobilis*	*Cryptococcus* sp., *Candida parapsilosis, Clavispora lusitaniae, Debaryomyces carsonii, Hanseniaspora uvarum, Kluyveromyces lactis, K. marxianus, Geotrichum candidum, Pichia* sp., *P. guilliermondii, P. membranifaciens, Rhodotorula* sp*., R. mucilaginosa, Saccharomyces bayanus, S. cerevisiae, S. pastorianus, Torulaspora delbrueckii*	Essential microorganisms responsible for lactic and acetic fermentation (LAB and acetic acid bacteria), alcoholic fermentation (*Z. mobilis* and *S. cerevisase*), EPS production y (*Leucocnostoc* sp.) and putrefactive bacteria	Lappe-Oliveras et al., 2008
Analysis of 16S rDNA clone libraries allowed to identify *Lactobacillus acidophilus, L. kefir, L. acetotolerans, L. hilgardii, L. plantarum, Leuconostoc mesenteroides* subsp. *mesenteroides, L. pseudomesenteroides, Acetobacter pomorum, Gluconobacter oxydans, Zymomonas mobilis, Flavobacterium jhonsonae, Hafnia alvei*		Homofermentative *L. acidophilus* was identified as the most abundant microorganism in three analyzed samples from different geographical origin, suggesting a possible essential role in lactic acid fermentation. *L. mesenteroides* was present in low proportion respect lactobacilli. *Z. mobilis* and AAB were detected low percentage or absent. Presence of possible putrefactive or contaminant bacteria	Escalante et al., 2004
A combined culture dependent and 16S rDNA libraries approach allowed to identify those microorganisms present in freshly collected *aguamiel* and during a 6 h of fermentation. α-Proteobaceria: *Acetobacter malorum*^a^, *A. orientalis*^b^, *Z. mobilis* subsp. *pomaceae*^b^, γ-Proteobacteria: *Citrobacter* sp., *Enterobacter* sp.^a^, *E. agglomerans*^a^, *Erwinia rhapontici*^a^, *Kuyvera acorbata*^c^, *K. cochleae*^a^, *Providencia* sp.^a^, *Serratia grimensii*^a^, *Acinetobacter radioresistens^b^, Sterotrophomonas* sp.^a^, *Chryseobacterium* sp. Firmicutes: *Bacillus* sp.^a^, *B. licheniformis*^a^, *Lactobacillus* sp.^c^, *L. acidophilus*^b^, *L. hilgardii*^b^, *L. paracollinoides*^b^, *L. sanfranciscensis*^b^, *Lactocoocus* sp.^a^, *L. lactis*^a^, *L. lactis* susp. *lactis*^a^ *Leuconostoc kimchi*^c^, *L. citreum*^c^, *L. gasocomitatum*^b^, *L. mesenteroides*^c^, *L. pseudomesenteroides*^c^, *Pediococcus urinaeequi*^a^, *Streptococcus deviesei*^a^	*S. cerevisiae*^b^	*Leuconostoc citreum* and *L. kimchi* species were identified as the most abundant LAB in *aguamiel*. After mixing fresh *aguamiel* with previously fermented *pulque, L. acidophilus, L. mesenteroides* were the most abundant LAB during 6 h of fermentation. *E. agglomerans* was the most abundant non-LAB during the first 3 h of fermentation. *Z. mobilis* and AAB were absent in *aguamiel* but detected in low proportion during the fermentation process Total bacterial counts (CFU/mL) for LAB and total aerobic mesophilic bacteria were constant during 6 h of fermentation. Total yeast counts (CFU/mL) detected in *aguamiel* increased after mixing *aguamiel* with fermented *pulque*, increased until 3 h and maintained constant until the end of the fermentation	Escalante et al., 2008

*(+) Indicates killer activity detected*.

a *Identified from a culture isolate*.

b *Identified from 16S rDNA clone library*.

c *Identified by culture and non-culture dependent approaches*.

The successful screening of the *aguaniel* and *pulque* for the isolation of diverse *Leuconostoc* and *Lactobacillus* species showing some *in vitro* and *in vivo* probiotic properties have been the subject of several reports (Table 4). These properties include:

- Resistance to antimicrobial barriers in the gastrointestinal tract such as lysozyme dilution by saliva, acid pH, gastric solution, and bile salt (Castro-Rodríguez et al., 2015; González-Vázquez et al., 2015; Giles-Gómez et al., 2016; Reyes-Naya et al., 2016; Torres-Maravilla et al., 2016).
- Antimicrobial activity against pathogenic bacteria such as enteropathogenic *Escherichia coli, Salmonella enterica* serovar Typhimurium, *S. enterica* serovar Typhi and *Listeria monocytogenes* (Castro-Rodríguez et al., 2015; González-Vázquez et al., 2015; Giles-Gómez et al., 2016; Torres-Maravilla et al., 2016).
- *In vivo* adherence to mice intestinal mucosa (Castro-Rodríguez et al., 2015).
- *In vivo* anti-inflammatory activity in a mouse model (Torres-Maravilla et al., 2016).
- *In vivo* anticholesterolemic affect (Reyes-Naya et al., 2016).
- *In vivo* anti-infective effect against *S. enterica* serovar Typhymurium (Giles-Gómez et al., 2016).

**Table 4:** **Probiotic assessment of LAB isolated from ***aguamiel*** and ***pulque*****.

**Source and identity of studied LAB**	**Resistance to *in vitro* gastrointestinal exposition conditions**	**Other relevant *in vitro* or *in vivo* activity**	**References**
*Lactobacillus brevis* isolated from *pulque*	This isolate strain showed 60% relative survival after acid exposition (pH 1.5), and 50–55% relative survival to simulated gastric acid exposition (pH 2.0). Bile tolerance to 0.3% taourocholic acid < 80%. Incubation conditions assayed: 4 h, 37°	Resistance to cefepime antibiotic, higher activity of bile salt hydrolase in MRS supplemented with 0.5% of taourocholic acid (671.72 U/mg protein)	González-Vázquez et al., 2015
*Leuconostoc mesenteroides* subsp. *mesenteroides* isolated from *aguamiel* (four strains)	Isolates showed < 50% survival to acid exposition (pH 2, 3 h, 37°C). Bile tolerance to 0.5% oxgall (4 h, 37°C)	All strains showed resistance to dicloxacillin, pefloxacin, trimethoprim, ceftazidime antibiotics. *In vitro* antimicrobial activity of cell-free supernatants against *Escherichia coli, Salmonella enterica* and *Listeria monocytogenes*. Bacterial adherence to mice intestinal mucosa	Castro-Rodríguez et al., 2015
*Lactobacillus plantarum, L. paracasei* subsp. *paracasei, L. brevis, L. composti, L. sanfranciscensis* isolated from *pulque* (14 isolates)	Two assayed strains showed >80% survival to lysozyme exposition. Three assayed strains showed > 80% survival to both acid pH (2.5) and 0.3% bile salts exposition. Exposition conditions assayed: 3 h, 37°C	Low binding capacity to HT-29 cells (~0.3%, best result) and to HT-29-MTX cells (10.78%, best result). In both assays, the binding capacity of isolated LAB was higher than control strain (*L. casei* BL23). Isolate identified as *L. sanfranciscensis* improve mice health by reduction of weight loss, significant decreases in gut permeability and anti-inflammatory effect by blocking the secretion of cytokines	Torres-Maravilla et al., 2016
*Lactobacillus brevis* and *L. collinoides* isolated from *aguamiel* (14 isolates)	Resistant to an *in vitro* model simulating gastrointestinal conditions	Capable of dissociating conjugated bile salts by the presence of diverse bile salt hydrolases. Some isolates were resistant to dicloxacillin, pefloxacin and ceftazidime antibiotics. The isolated strain of *L. brevis* Lb9H showed *in vivo* protective effect of liver damage associated with the prevention of ALT^a^ activity and preventing the intoxication by LPS+D-GalN^b^, indicator of lipid peroxidation	Reyes-Naya et al., 2016
*L. mesentreoides* strain P45 isolated from *pulque*	Resistance to lysozyme exposition 70% (2 h, 37°C). 100% resistance to 0.3% and 1% bile salts exposition (4 h, 37°C). ~75% resistance to acid exposition (pH 2.5, 5 h, 37°C). This strain showed remarkable resistance to combined acid (pH 2.5) and bile salt (0.3%) exposition for 24 h, 37°C	*In vitro* antimicrobial activity against enteropathogenic *E. coli, S. enterica* serovar Typimurium, *S. enterica* serovar Typhi and *L. monocytogenes* in cell-to-cell assays (LAB-pathogen), cell-free supernatants assays and EPS-producing cell-to-cell assays (LAB-pathogen). *In vivo* assays showed that administration of strain P45 is associated with an important decrement in *S. enterica* serovar Typhimurium infection in liver and spleen in BALB/c female and male mice	Giles-Gómez et al., 2016

a *Serum alanine transferase*.

b *Lipopolysaccharide + D-Galactosamine*.

This scientific evidence of LAB responsibility for health-promoting effects associated with *pulque* consumption makes these bacteria relevant probiotic candidates for the development of non-dairy based functional products.

### Functional properties of EPS produced by LAB detected in *aguamiel* and *pulque*

Some EPS produced by LAB isolated from *aguamiel* and *pulque* have been purified and characterized. Results include the identification of dextran with a linear backbone linked in α1 → 6 D-Glc*p* linkages with branching in α1 → 3 D-Glc*p* produced by a cell-associated glycosyltransferase (GTF) from *L. mesenteroides* isolated from *pulque* collected from the Apan region, in the state of Hidalgo (Chellapandian et al., 1998). In the same context, two EPS LAB identified as *L. kimchii* were isolated from *pulque* produced in Huitzilac, in the state of Morelos. One of the strains (EPSA) produced dextran with a linear backbone joined by α1 → 6 D-Glc*p* with α1 → 2 and α1 → 3 branching linkages through enzymes found in the soluble and the cell-associated fractions. The second strain (EPSB) produced a polymer mixture including a levan composed by linear chains containing β2 → 6 linked β-D-fructofuranosyl moieties and β2 → 1 branches (79%), as well as a dextran Type I polymer (21%) (Torres-Rodríguez et al., 2014).

EPS and hetero-oligosaccharides produced by diverse LAB species, including those found in *pulque*, have gained attention because of their use as food additives and potential natural functional ingredients. Their main applications include their use as prebiotic agents as well as soluble fiber (Patel et al., 2011; Harutoshi, 2013) such as those produced by *Lactobacillus reuteri, L. rhamnosus, L. acidophilus*, and *Bifidobacterium bifidum* (Helal et al., 2015). EPS produced by LAB with potential probiotic properties have been proposed to play a positive effect in the intestinal adhesion (García-Ruiz et al., 2014). *In vitro* antimicrobial assays with EPS-producing *L. mesenteroides* strain P45 isolated from *pulque* against EPEC *E. coli, S. enterica* serovar Typhimurium, *S. enterica* serovar Typhi, and *L. monocytogenes* showed an improved *in vitro* antimicrobial activity in EPS-producing cell-to-cell assays (Giles-Gómez et al., 2016). These results are preliminary, as the detailed mechanisms involved both *in vivo* and *in-vitro* potential functional properties of EPS produced by LAB, particularly those species assayed for potential probiotic activities remain to be determined.

## *Pulque* industrialization and major technological challenges

### Science and technology of *pulque*

A simple look at research figures illustrates the lack of interest in *pulque* by the scientific community: A PubMed search under “beer” results in today in 17,929 hits while only 30 references come out under “*pulque*” most of them published in the twenty-first century. However, 8 of them were released in the last 2 years (2014 and 2015) as evidence of a renew interest.

It is worthwhile looking at this extremely low figure in more detail, as the earliest scientific publication dealing with the process, dates back to 1957 when Alfredo Sanchez Marroquin (Sánchez-Marroquín et al., 1957), first tried to industrialize *pulque* starting from the basic/minimum microbiological requirements to transform *aguamiel* into *pulque*. We, of course, acknowledge the initial efforts of Dr. Leopoldo Río de la Loza to elucidate the microbiology of *pulque* in 1864. He reported in the *Boletin de la Sociedad de Geografía y Estadística*, the isolation of *Termobacterium mobile* by Paul Lindner in 1924 (Weir, 2016), among others. *Pulque*'s microbiology, the isolation of strains, and more recently, its individual probiotic characterization, is probably the main research trend (Torres-Rodríguez et al., 2014; Castro-Rodríguez et al., 2015; González-Vázquez et al., 2015; Giles-Gómez et al., 2016; Torres-Maravilla et al., 2016). An additional research subject deals with the effect of *pulque* in the Mexican diet. The first reference given by PubMed is a document from 1897 in which Francisco Martínez Baca, a famous physician from the state of Puebla described the successful treatment with *pulque* of penitentiary inmates suffering scurvy (published in the Journal of the American Public Health Association) (Ramírez Rancaño, 2000). It was not until 1933 that vitamin C was finally discovered (Carpenter, 2012).

However, no references deal with *pulque* production technology, scaling up of the process, neither the definition of the main microbiota required to reproduce the beverage, as consumers know it. These concerns remain as technological challenges since last century when Sanchez Marroquin defined the four physiological processes involved in *pulque* production (Sánchez-Marroquín et al., 1957). Nevertheless, reducing the microbiota to three or four microorganisms would blindly eliminate possible bacteria contributing as probiotics to the claimed beneficial health effects, particularly to treat gastrointestinal problems and diarrhea. The simple decision between *S. cerevisiae* or *Z. mobilis* as the alcohol producer is not that evident. *S. cerevisiae* reaches higher ethanol concentrations without inhibition, while *Z. mobilis*, a faster ethanol producer, also contains two levansucrases, responsible for levan synthesis, part of the soluble fiber in which *pulque* is particularly rich (Lau et al., 2010; Xiong He et al., 2014; Weir, 2016). Up to now, *pulqu*e remains as a very heterogeneous beverage regarding its common final organoleptic properties (alcohol-acid taste and viscosity): while many drinkers prefer the fresh product, others prefer *pulque* after more than 24 h of fermentation combined with fruit juice (*curados*). Nevertheless, *pulque* does not stand large storage times without developing off flavors, and pasteurization not only affects flavor but also destroys one of its main properties: the microbiota.

It is probably to this aspect that the largest (but still minor) efforts in research have been devoted. The presence of prebiotic fructooligosaccharides from agave inulin present in *aguamiel*, as well as the soluble inulin-like agavin, levan and dextran polysaccharides have been described and characterized (Chellapandian et al., 1998; Ortiz-Basurto et al., 2008; Torres-Rodríguez et al., 2014). Some of this prebiotics have been evaluated both *in vitro* and *in vivo*, and we suggest that the beneficial effects observed among lactating mothers and their babies (Argote-Espinosa et al., 1992; Backstrand et al., 2001, 2004) is mainly due to its pre- and probiotic content. Unfortunately, most research is now devoted to the isolation and production of probiotic bacteria as alternative beverages, isolated from *pulque*, but out of the scope of the beverage. These efforts are similar to those carried out last century by Paul Lindner himself. He was convinced that *Pseudomonas lindneri* (that he had previously defined as *Thermobacterium mobile*) was responsible for the beneficial effects of *pulque* in the treatment of intestinal disorders and produced in Berlin from this single bacteria a “functional” fermented beverage (Gonçalves de Lima, 1956).

### Challenges associated with *pulque* production

Probably the main challenge associated with the industrialization of *pulque* is related to the natural substrate availability and the need for the introduction of a stabilization processes of the fermented product. *Aguamiel* differs from almost all other fermented beverages such as wine, beer or *tepache* (pineapple wine), in that agave, the raw material, takes 7 years to reach maturity. Furthermore, when ready for production, *aguamiel* has to be collected from the plant on a daily basis, and not produced by a single extraction, as it is usually the case for fermented beverages. Each agave plant is visited daily during several months and the accumulated *aguamiel* extracted, a labor-intensive activity, which also induce fermentation in the plant itself where *aguamiel* accumulates during the day. Therefore, the fermentation is already taking place when the substrate is collected. In contrast, the fermentation that leads to tequila or mezcal, also produced from agave sugars, does not require this process as sugars are extracted directly from the mature plant (*Agave tequilana*) in a single operation after the agave pine is cooked and mashed.

Several successful efforts for industrialization for the production of bottled/canned fermented *pulque* have been performed mainly by producers in the States of Puebla, Tlaxcala and Hidalgo (Ramírez et al., 2004; Jaurez Rosas, 2015). The producers include companies as Tecnología e Innovación en *Pulque* Industrial S.A. de C.V., comprising more than 300 *pulque* producers in Puebla state, Torre Grande in Hidalgo and Procesadora de *Pulque* S.A. de C.V and *Pulque* Hacienda 1881 in Tlaxcala. Both companies export canned *pulque* to Europe, Central America, and the United States, the latter being the largest market for canned *pulque* (mainly the cities of Los Angeles and Chicago where are the biggest settlement of Mexican immigrants) (Jaurez Rosas, 2015). However, the industrialization of *pulque* introduced fundamental changes in the public perception of traditional producers and consumers resulting in a product that the majority of traditional consumers never tasted before. Efforts to stabilize the fermented beverage by pasteurizing and/or filtrate *pulque* or by the addition of preservatives, antioxidants, colorants or texturizing agents will certainly improve stability and shelf life but could reduce the pre- and probiotic content of the fermented beverage (Ramírez et al., 2004; Escalante et al., 2012).

However, there is an increasing preference for local products and local markets (Jaurez Rosas, 2015). We believe that the main scientific and technological investment should come from the demonstration of the main nutritional, health-promoting and organoleptic attributes of *pulque* and its microbiota, introducing specific modifications in the traditional production *tinacales* that bring assurance to the consumer that *pulque* is produced hygienically, conserving its local characteristics and its regular strains, but safe to the consumer.

### Functional genomics of *pulque* and relevant microorganisms involved in the fermentation process

Application of a culture-independent approach such as 16S rDNA clone library to the study of bacterial diversity present in *aguamiel* and *pulque* allowed to determine a remarkable LAB diversity, suggesting an essential role of these microorganisms in the *fermentation* process (Escalante et al., 2004, 2008). Emerging research on the microbiology of *pulque* focuses on the isolation and *in vitro* as *in vivo* assessment of probiotic LAB with promising capabilities (Castro-Rodríguez et al., 2015; González-Vázquez et al., 2015; Giles-Gómez et al., 2016; Reyes-Naya et al., 2016; Torres-Maravilla et al., 2016; Table 4).

Functional genomics from available LAB genome information has provided new insights regarding the evolution of LAB, their metabolic profile and the interactions with other microorganisms and the environment, allowing to understand the role of these microorganisms in traditional or industrial food fermentations and their interactions with the human hosts (Douillard and de Vos, 2014). Genome sequencing of relevant LAB isolated from *pulque*, such as those recently identified with potential probiotic properties promises to provide valuable information on the genetic traits involved in the probiotic activity.

Complete genome analysis of potential probiotic *L. mesenteroides* strain P45 by Riveros-McKay et al. (2014), allowed the identification of diverse genes probably involve in the antimicrobial activity of this LAB such as those coding for diverse peptidoglycan hydrolases and a prebacteriocin (Giles-Gómez et al., 2016). This information provides new insights to focus further efforts on the characterization of the potential probiotic of this LAB from *pulque*. However, the next step in the study of *pulque* microbiology relies on the application of metagenomic approaches to study the entire microbial composition (including both bacteria and yeasts) in combination with other high-throughput omic methodologies such as transcriptomics, metabolomics or proteomics. These approaches applied to other regional traditional fermented foods and beverages (e.g., Korean *kimchi* Jung et al., 2011), could provide valuable insights into the complex microbial community involved in the fermentation process.

## Concluding remarks

All through Mexican history, from pre-hispanic times to our days, *pulque* has been a key reference regarding culture, tradition, and cuisine. Once the center of the cosmological vision of our ancestors, later a source of wealth through agro-industrial exploitation, abandoned and despised -described as a nutrient of underdevelopment and ignorance after the Revolution Civil War, and now the subject of wonder and scientific research. *Pulque* is now the center of research in many laboratories, not only due to its nutritional properties but also to the extremely complex microbial diversity responsible for its fermentation, a process that has resisted industrialization. No doubt, *pulque* is an essential element for the UNESCO decision in 2010 to include the traditional Mexican cuisine in the List of the Intangible Cultural Heritage of Humanity.

## Author contributions

DL and JV collected the video and photographic material included in this contribution and prepared the information corresponding to the traditional process of *pulque* fermentation. AE, MG, FB, and AL wrote the manuscript and designed the graphic material. All the authors reviewed and approved the final version of the manuscript.

## Funding

This contribution was supported by Programa de Apoyo a Proyectos de Investigación e Innovación Tecnológica (PAPIIT), UNAM project IN207914.

### Conflict of interest statement

The authors declare that the research was conducted in the absence of any commercial or financial relationships that could be construed as a potential conflict of interest.

## Supplementary material

Supplementary File 1 **Castration process of a mature maguey for *aguamiel* production 1**. (Video in mp4 format, 2:27 min). *Pulque* producer or *tlachiquero* perform castration process. Once the plant has been selected the *tlachiquero* prepares the maguey by cutting off the central leaves of the plant surrounding the floral bud with a sharpened knife. With the floral peduncle exposed (“opening the door”), the *tlachiquero* cut off this part of the plant with a knife.

Supplementary File 2 **Castration process of a mature maguey for *aguamiel* production 2**. (Video in mp4 format, 2:03 min). The remaining floral bud is destroyed to avoid the possible development of the embryonic floral peduncle. For this operation, the *tlachiquero* uses a pointed and sharpen metallic instrument (a jimmy bar) to make a pit in the residual floral bud (0:00–0:43 min). Finally, the *tlachiquero* uses a scraping tool to make the final shape of the cavity (*cajete*) and covers the pit with a maguey leaf (0:43–2:03 min).
